# Comparing High-Flow Nasal Cannula and Non-Invasive Ventilation in Critical Care: Insights from Deep Counterfactual Inference

**DOI:** 10.21203/rs.3.rs-7230866/v1

**Published:** 2025-08-05

**Authors:** Xiaolei Lu, Michael Miller, Alex K. Pearce, Jonathan Y. Lam, Aaron E. Boussina, Kai Zheng, Atul Malhotra, Shamim Nemati

**Affiliations:** Department of Biomedical Informatics (Xiaolei Lu, Jonathan Y. Lam, Aaron E. Boussina, and Shamim Nemati), the Division of Pulmonary, Critical Care, and Sleep Medicine (Michael Miller, Alex K. Pearce, Atul Malhotra), University of California, San Diego, La Jolla, CA; Department of Informatics (Kai Zheng), University of California, Irvine, Irvine, CA

**Keywords:** Individualized Treatment, Counterfactual Modeling, Invasive Mechanical Ventilation, High-Flow Nasal Cannula, Noninvasive Ventilation

## Abstract

Randomized trials comparing high-flow nasal cannula (HFNC) and non-invasive positive pressure ventilation (NIV) for acute respiratory failure (ARF) offer population-level guidance but often fail to capture individual variability in treatment response. In this retrospective study, we identified intensive care units (ICU) patients at risk of invasive mechanical ventilation (IMV) using a previously published risk prediction model. Patients who first received HFNC or NIV after crossing the high-risk threshold formed the early treatment cohort. We developed a deep counterfactual model that integrates representation learning, conditional normalizing flows, and confounder adjustment to estimate individualized treatment effects (ITEs) between HFNC and NIV. Treatment concordance, defined as alignment between the model’s recommendation and the treatment actually administered, was assessed using multivariate logistic regression. At UC San Diego Health (UCSD), concordant treatment was associated with significantly reduced odds of IMV (odds ratio [OR] = 0.661 for NIV; 0.677 for HFNC) and mortality or hospice discharge (OR = 0.679 for NIV; 0.749 for HFNC). At UC Irvine Health (UCI), concordant treatment was also linked to improved outcomes, particularly for mortality or hospice discharge (OR = 0.092 for NIV; 0.088 for HFNC). These findings highlight the value of individualized, model-guided respiratory support strategies in improving outcomes for critically ill patients.

## Introduction

Acute respiratory failure (ARF) is a common reason for hospitalization, both in general wards and intensive care units (ICU). Oxygen therapy, generally with simple nasal cannula, is often the initial treatment modality for those patients with hypoxemia. If respiratory failure progresses, and patients require more than 6–8L/min of oxygen to maintain adequate oxygen saturation or exhibit respiratory distress or hypercarbia, treating physicians escalate respiratory support in several different way including high flow nasal cannula (HFNC; airflows up to 50–60L/min), non-invasive positive pressure ventilation (NIV), or invasive mechanical ventilation (IMV). While the choice of the initial treatment modality is important and could influence patient outcomes, there remains clinical equipoise surrounding this treatment decision as several randomized controlled trials have shown mixed results.[2–11]

Medical society guidelines provide general guidance and advice for some specific clinical contexts. For example, the European Respiratory Society (ERS) and American Thoracic Society (ATS) guidelines[12] support NIV use for acute exacerbation of chronic obstructive pulmonary disease (AECOPD), cardiogenic pulmonary edema, immunocompromised patients with ARF, post-operative respiratory failure, palliative treatment of dyspnea, chest trauma patients with ARF, and preventing post-extubation respiratory failure in high-risk patients. However, the guidelines do not make recommendations about asthma, de novo ARF, or pandemic viral illness. Guideline updates from the ERS in 2022[13] added that patients with acute hypoxemia respiratory failure should generally be treated with HFNC over NIV, HFNC or NIV is equivocal for post-operative patients at high-risk of respiratory complications, and removed the recommendation for NIV use in immunocompromised patients. Many of these recommendations are conditional with low certainty of evidence. Real-world clinical care is often complicated, and patients can have multiple co-occurring conditions with varying indications or contraindications (e.g., multifocal pneumonia, pulmonary edema and AECOPD, neuromuscular weakness, facial trauma or surgery, large beards, inability to manage secretions or protect airway, severe encephalopathy, hemodynamic instability, gastric or esophageal pathology, etc.) for either HFNC or NIV.

Another feature complicating decisions regarding oxygen delivery modality is that ICU patients themselves are often inhomogeneous and many of the conditions commonly encountered in the ICU (sepsis, ARF, etc.) are syndromes composed of a variety of pathophysiologic states and severities. Controlling a myriad of disease states and patient-specific differences in randomized clinical trials in this population is difficult. It is therefore unsurprising that trials in this arena commonly fail to demonstrate a difference between treatment and control groups, show mixed results among studies, or fail to provide generalizable results. One explanation for these mixed findings is that the average treatment effect (ATE) captured by randomized control trials (RCTs) may hide variations of the treatment’s effect on a clinical outcome across levels of patient characteristics. Another way to rephrase is that even with a null overall result, due to heterogeneity of treatment effect (HTE), some patients may benefit from an intervention, others are unchanged, and others may even be hurt.

Individualized treatment effect (ITE) predictive modeling provides patient-specific estimates of treatment response to capture heterogeneity in how patients respond to interventions. Within the area of machine learning (ML), counterfactual models, often applied to observational data, have been developed within the potential outcome framework to estimate the outcomes of different treatment strategies by simulating “what-if” scenarios.[14–17] Deep counterfactual models build on this idea by incorporating complex clinical data and adjusting for measured confounding biases, such as patient demographics, comorbidities, prior treatments, and disease progression, to provide more accurate estimates of treatment effects.[18,19] By generating these counterfactual predictions, ML can aid clinicians to identify which therapies are most likely to benefit individual patients. However, most counterfactual models fail to account for unmeasured confounders, including clinical decision-making biases and hidden patient severity factors that influence treatment decisions. Additionally, the process of representation learning in deep counterfactual models can lead to the loss of important measured confounders,[20] which further impacts the ITE estimation.

We conducted a retrospective analysis of ICU patients at risk of progressing to IMV identified using the Vent.io respiratory failure risk prediction model.[1] To estimate the ITE of HFNC versus NIV as the initial respiratory support, we developed a deep counterfactual model RepFlow-CFR, a flow-based confounder adjustment framework that integrates representation learning, normalizing flows and counterfactual inference, which was designed to account for both measured and unmeasured confounders while improving causal effect estimation. We tested the hypothesis that the deep counterfactual model 1) would accurately identify the patients who would benefit from either HFNC or NIV, and 2) concordance between the model’s recommendations and the actual treatment administered is associated with improved clinical outcomes.

## Results

In this section, we first identified high-risk patients using the Vent.io model and defined the early HFNC/NIV cohorts at two academic ICUs. We then evaluated the predictive performance of our RepFlow-CFR model in forecasting the need for IMV, both before and after site-specific fine-tuning. Next, we analyzed ITE to categorize patients by likely benefit from HFNC or NIV, and assessed differences in clinical characteristics and outcomes across groups. We further examined the top features contributing to ITE predictions using SHAP analysis. Finally, we evaluated whether concordance between model-recommended and actual treatments was associated with improved clinical outcomes, using both descriptive statistics and multivariable regression models across two sites.

### Identification of Early HFNC/NIV Cohort

UCSD ICU cohort consists of 31,180 encounters, and UCI ICU cohort includes 3,290 encounters. Using the pretrained Vent.io, which was developed based on UCSD ICU cohort (Supplementary 5), we identified 5,685 encounters for UCSD high-risk cohort and 578 encounters for UCI high-risk cohort. The Vent.io achieved an AUC of 0.895, sensitivity of 0.603, specificity of 0.845 and positive predictive value of 0.177 at UCSD and an AUC of 0.842, sensitivity of 0.547, specificity of 0.836 and positive predictive value of 0.167 at UCI. Patient characteristics for these two ICU cohorts are reported in Table S5. We then filtered and selected patients who meet the criteria of first receiving either HFNC or NIV after Vent.io T0, with 1,956 encounters in UCSD early HFNC/NIV cohort and 169 encounters in UCI early HFNC/NIV cohort. Table 1 shows baseline characteristics in UCSD and UCI early HFNC/NIV cohorts. NIV-treated group has a higher Charlson Comorbidity Index (CCI) score and a higher prevalence of Chronic Obstructive Pulmonary Disease (COPD) and Congestive Heart Failure (CHF) compared to the HFNC-treated group at both sites.

### IMV Predictive Performance

RepFlow-CFR achieved an AUC of 0.820 and a PR-AUC of 0.566 on the UCSD early HFNC/NIV cohort, which is comparable to the baseline CFR model (AUC: 0.821, PR-AUC: 0.571). However, when externally validated on the UCI early HFNC/NIV cohort, performance declined, with RepFlow-CFR achieving an AUC of 0.630 and a PR-AUC of 0.444, compared to CFR’s AUC of 0.656 and PR-AUC of 0.415. To improve generalizability, we fine-tuned both CFR and RepFlow-CFR using 25% of the UCI early HFNC/NIV cohort. After fine-tuning, RepFlow-CFR achieved an AUC of 0.727 and a PR-AUC of 0.553, while CFR achieved an AUC of 0.758 and a PR-AUC of 0.590 on the UCI site.

### Predicted Individualized Treatment Effects

To categorize patients based on their predicted ITE, we defined absolute ITE ≤ 0.001 as indicating patients who are non-responders or indifferent to both treatments despite minor fluctuations in ITE estimates, and divided the patients into three groups: NIV preferred (ITE < −0.001), HFNC preferred (ITE > 0.001), or Indifferent (ITE between −0.001 and 0.001). Table 2 summarizes the characteristics of patients by the predicted ITE of RepFlow-CFR model. At UCSD, the HFNC preferred group exhibited the highest CCI and showed a greater prevalence of CHF and COPD compared to the NIV preferred group. Baseline physiological measures, including heart rate, respiratory rate, temperature, mean arterial pressure, and PaCO2, did not differ significantly across groups at either site. Rates of IMV were highest in the NIV preferred group at both UCSD (25.2%) and UCI (37.9%). Meanwhile, mortality and hospice outcomes were not significantly different across treatment groups at either site. The directionality of average treatment effects was maintained across both cohorts. While traditional group analysis may overlook important individual differences, the ITE identifies these variations to have more personalized treatment recommendations.

### SHAP-Based Feature Interpretation of ITE Predictions

**Fig. 1:**
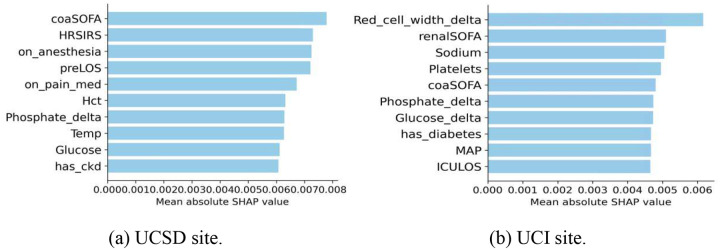
Top 10 features ranked by mean absolute SHAP value for ITE prediction using the RepFlow-CFR model. (a) UCSD site. (b) UCI site.

To better understand the ITE predictions made by the RepFlow-CFR model, we applied SHAP values to quantify the contribution of each input feature. Figure 1 presents the top 10 features ranked by mean absolute SHAP value for the UCSD and UCI cohorts. At the UCSD site (Figure 1a), the most influential features included severity scores (e.g., coaSOFA, HRSIRS), pre-hospital factors (e.g., preLOS), and clinical interventions such as anesthesia and pain medication use, suggesting that patient acuity and treatment context strongly influenced individualized treatment recommendations. At the UCI site (Figure 1b), laboratory values and organ-specific dysfunction, particularly Red_cell_width_delta, renalSOFA, and Sodium, were more dominant, indicating site-specific variation in model interpretation. While some features such as coaSOFA and Phosphate_delta appeared in both cohorts, the overall differences underscore the importance of local data characteristics in shaping treatment effect predictions.

### Outcomes with treatment concordance

Our primary clinical outcome was the need for IMV, and our main secondary outcome was combination of in-hospital mortality and disposition to hospice. We first computed the outcome proportions based on whether patients received treatments in concordance with the ITE-predicted preferred treatment NIV or HFNC according to each method. By comparing concordance versus discordance groups across different ITE estimation approaches, we can assess whether aligning treatment decisions with model recommendations leads to improved outcomes.

**Fig. 2:**
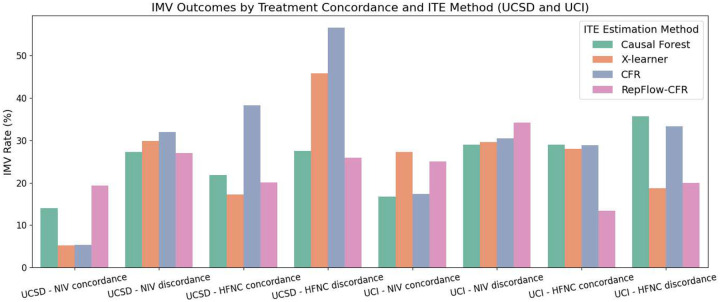
Comparison of IMV outcomes by treatment concordance and ITE estimation method. Bars represent IMV rates across four treatment groups (NIV concordance, NIV discordance, HFNC concordance, HFNC discordance) for each ITE method.

**Fig. 3:**
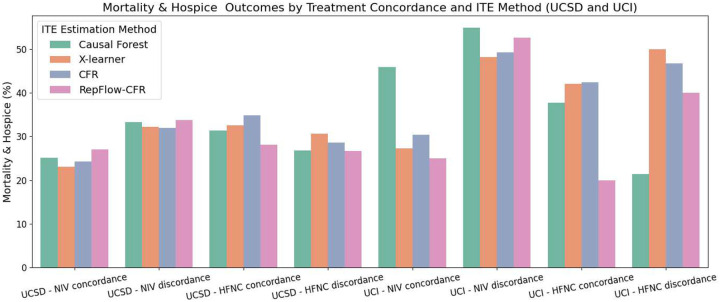
Comparison of Mortality & Hospice outcomes by treatment concordance and ITE estimation method. Bars represent Mortality & Hospice rates across four treatment groups (NIV concordance, NIV discordance, HFNC concordance, HFNC discordance) for each ITE method.

As illustrated in Figures 2 and 3, both IMV and Mortality & Hospice outcomes were generally more favorable for patients who received treatments concordant with their predicted benefit across both sites. For IMV, RepFlow-CFR consistently showed lower rates in the concordant groups, including 19.27% vs. 27.06% for NIV at UCSD, and 13.33% vs. 20.00% for HFNC at UCI. Most methods followed similar trends, although the X-learner at UCI showed slightly higher IMV in the HFNC-concordant group. Mortality & Hospice outcomes also reflected this concordance benefit: RepFlow-CFR showed 27.22% vs. 33.72% for NIV at UCSD, and 20.00% vs. 40.00% for HFNC at UCI. Overall, concordant treatment was associated with better outcomes, with RepFlow-CFR demonstrating the most consistent improvements across settings and outcomes.

To evaluate whether concordance with the predicted treatment is independently associated with improved clinical outcomes, we developed two multivariable logistic regression models: one for predicting the need for IMV, and another for predicting a composite outcome of mortality or hospice. Both models adjusted for key clinical confounders, including age, gender, SOFA score, CCI score, and Vent.io score. As shown in Table 3, concordance with the treatment recommended by RepFlow-CFR was significantly associated with reduced risk of IMV at the UCSD site for both NIV and HFNC. Similarly, in the composite outcome model, RepFlow-CFR showed the strongest effect at the UCI site, with both NIV concordance and HFNC concordance significantly associated with lower mortality or hospice. These associations were not consistently observed with baseline methods such as Causal Forest, X-learner, or CFR. While some alternative models showed significant results at one site or for one treatment, RepFlow-CFR was the only method demonstrating robust and consistent benefit across both outcomes and treatment modalities.

## Discussion

In this study, we developed and validated a deep counterfactual model, RepFlow-CFR, to estimate individualized treatment effects (ITE) of HFNC versus NIV as initial respiratory support for critically ill ICU patients at risk of requiring IMV. Our findings demonstrate that personalized treatment recommendations based on model-predicted ITEs were associated with improved clinical outcomes, including reduced rates of IMV and a combined endpoint of mortality or hospice.

These results underscore the limitations of relying solely on average treatment effects from randomized controlled trials (RCTs), which may obscure important heterogeneity in individual responses to therapy. While clinical guidelines provide important direction for specific patient population, such as those with acute exacerbation of COPD or cardiogenic pulmonary edema, many ICU patients present with complex and overlapping clinical features not addressed by these guidelines. As such, treatment decisions often rely on physician judgment, which may be subject to variability and bias.

By leveraging deep learning techniques, representation learning, and conditional normalizing flows, RepFlow-CFR offers a novel approach to estimate counterfactual outcomes while adjusting for both measured and unmeasured confounders. Treatment concordance with model-predicted recommendations was significantly associated with improved outcomes at both development and validation sites, suggesting that individualized treatment guidance could play a meaningful role in clinical decision-making. Compared to traditional ITE estimation methods such as Causal Forest, X-Learner, and baseline CFR, our model demonstrated more robust and consistent performance, particularly in the external cohort after fine-tuning.

The model’s performance in the validation cohort also demonstrates the feasibility of adapting counterfactual models to diverse patient populations with limited retraining. This is critical for real-world implementation, where patient characteristics, comorbidity profiles, and practice patterns may differ substantially across institutions. Moreover, the ability of RepFlow-CFR to integrate fine-grained clinical variables and learn nuanced patient representations positions it as a powerful tool for personalized critical care.

Importantly, our findings suggest that personalized, data-driven decision support could be complementary to existing guideline-based care. In cases where guidelines are ambiguous or do not fully account for patient complexity, counterfactual models may offer more precise treatment selection and help avoid unnecessary escalation or delay in care. Future research could focus on hybrid approaches that combine model-derived ITEs with clinical expertise and guideline constraints to develop interpretable, actionable tools for bedside decision-making.[24]

However, several limitations must be acknowledged. As a retrospective analysis, our findings are subject to biases inherent in observational data, including residual confounding due to unmeasured variables such as clinician intent or undocumented clinical indicators. Although we attempted to account for such biases using advanced modeling techniques, prospective validation is needed to establish causal conclusions. Additionally, both study sites were academic centers in the same geographic region, and further validation in community and international settings would enhance generalizability.

## Methods

### Study Design and Setting

We conducted a retrospective study using de-identified Electronic Health Record (EHR) data of all adult patients (≥18 years) who were admitted to the ICU at University of California San Diego Health (UCSD) between January 1, 2016, and December 31, 2023, and the University of California Irvine Health (UCI) between January 1, 2021, and August 15, 2022, as well as January 1, 2023, and August 31, 2024. Ethics approval was obtained from the University of California San Deigo Institutional Review Board.

Patients were included in the respiratory failure prediction analysis if they had an ICU stay of at least five hours, were not mechanically ventilated before ICU admission, and had recorded measurements of heart rate, blood pressure, and laboratory values prior to the prediction start time. If there were multiple admissions for a single patient, each ICU stay was treated as a separate encounter. Patients with a “Do Not Resuscitate” (DNR) order were excluded, and timestamps within 24 hours before and after surgery were omitted to avoid capturing surgery-related ventilation events. Monitoring for respiratory failure continued throughout each ICU stay until either mechanical ventilation was initiated or the patient was transferred out of the ICU. To ensure sufficient data collection, predictions began four hours after ICU admission and were updated hourly using the latest clinical data.

We used the pretrained Vent.io respiratory failure risk prediction model to identify high-risk ICU patients by thresholding the predicted state value. The model was trained using a custom 5-point labeling scale for mechanical ventilation (Table S1) to account for the various physiological states of respiratory failure, where a score ≥ 3 was defined as positive class and < 3 was control class. The high-risk threshold was selected at 60% sensitivity at the encounter level of the training data, based on clinical feedback, to minimize false positives. This threshold was then applied during model inference: if the predicted score met or exceeded the threshold, the patient was classified as high-risk for requiring IMV within the next 24 hours. A score below the threshold indicated that IMV was not predicted within the next 24 hours.

**Fig. 4:**
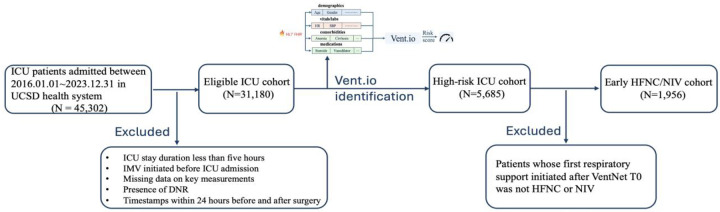
Cohort selection flowchart for early HFNC/NIV analysis (UCSD cohort shown as an example).

To evaluate the effect of initial treatment of HFNC and NIV on high-risk ICU patients, we defined Vent.io T0 as the time at which the patient’s risk score first crossed the high-risk threshold (score ≥ 3). We focused on the clinical data at Vent.io T0 and the initial respiratory support treatment administered after this timepoint, specifically including patients who first received either HFNC or NIV (referred to as early HFNC/NIV cohort). Treatment categories were further refined to distinguish specific HFNC and NIV interventions (Table S2). Figure 4 illustrates the cohort derivation process for the analysis of early HFNC and NIV as initial respiratory support following Vent.io-predicted high-risk timepoint (Vent.io T0). Using counterfactual modeling, we aimed to compare the effects of HFNC and NIV on the subsequent need for IMV and mortality.

### Data Abstraction, Missingness, and Processing

We extracted data from EHR including 50 vital signs and laboratory measurements, 6 demographic features, 12 Systemic Inflammatory Response Syndrome (SIRS) and Sequential Organ Failure Assessment (SOFA) criteria, 12 medication categories, and 62 comorbidities (Table S3). The vital signs and laboratory measurements were grouped into one-hour time series bins to account for varying data sampling frequencies. Variables sampled more than once per hour were resampled into hourly bins using their median. Updates were made hourly with new data and if no new data were present, existing values were carried forward for up to 24 hours. All remaining missing values were replaced using mean imputation. We reported missing data on an hourly interval following the resampling process for the 50 vital signs and laboratory measurements (Table S4). In addition to the above clinical variables, we calculated 150 features derived from the 50 vital signs and laboratory measurements. For each vital sign and laboratory measurement, we derived baseline values (mean value measured over the previous 72 hours), local trends (change since last measurement), and the time since the variable was last measured (TSLM).

### Model Development, Training and Evaluation

**Fig. 5:**
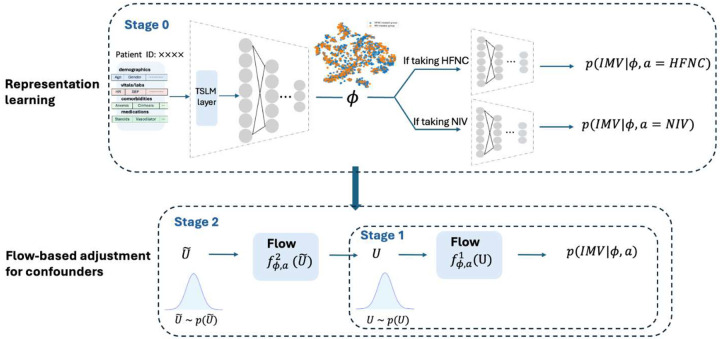
Overview of the RepFlow-CFR model, which contains CFR-based representation learning, output distribution modeling and hidden confounding adjustment.

We proposed the RepFlow-CFR model, a flow-based confounder adjustment model that integrates representation learning, normalizing flows and counterfactual inference. Figure 5 presents the architecture overview of the RepFlow-CFR model, which contains three modules as follows.

Stage 0 (CFR-based representation learning): We utilized the counterfactual regression architecture (CFR)[18] that includes shared representation layers and two distinct heads for predicting outcomes under different treatments. The shared representation layers, based on the Vent.io architecture, include a TSLM layer for adjusting the importance of labs and vitals, followed by a feedforward neural network. By training the CFR model, the shared representation is encouraged to balance the distribution of measured confounders across treatment groups. The loss function is formulated as

$$L_{0}=E\left[L_{\text{CFR}}(x,a,y)+\lambda \cdot IPM_{G}\left({\left\{\phi_{i}\right\}}_{i,a=\text{HFNC}},{\left\{\phi_{i}\right\}}_{i,a=\text{NIV}}\right)\right],$$

where *IPM*_*G*_ is the empirical probability metric (e.g. Wasserstein distance). *L*_*CFR*_ is the prediction loss. *λ* denotes the trade-off parameter that balances prediction accuracy and representation distribution matching.

After training Stage 0, we assume that the learned representation *ϕ* captures sufficient information from measured covariates such that potential outcomes are conditionally independent of treatment assignment, given *ϕ* and a latent variable *u* representing unmeasured confounding as

Y(A)╨A∣ϕ,u,

where *Y* and *A* denote the outcome and treatment, respectively.

The observed distribution is defined as

p(y∣ϕ,a)=∫p(y∣ϕ,u,a)p(u∣ϕ,a)du.

The interventional distribution, which removes the influence of *a* on *u*, is

p(Y(a)=y∣ϕ)=∫p(y∣ϕ,u,a)p(u∣ϕ)du.

If *p*(*u*|*ϕ*, *a*) = *p*(*u*|*ϕ*), we will have *p*(*Y*(*a*) = *y*|*ϕ*) = *p*(*y*|*ϕ*, *a*). However, in observational studies, this assumption rarely holds due to treatment assignment bias or unmeasured confounding. Directly using *p*(*y*|*ϕ*, *a*) to estimate counterfactual outcomes would lead to biased inference. To address this, RepFlow-CFR includes two additional stages to explicitly model and account for this hidden bias.

Stage 1 (Modeling outcome distribution): We used a conditional normalizing flow (CNF)[21] to model the observed outcome distribution *p*(*y*|*ϕ*, *a*), where *a* ∈ {*NIV*, *HFNC*}. The CNF learns an invertible transformation $f_{\phi ,\text{a}}^{1}$ that maps a standard normal latent variable *U*~*N*(0, *I*) to the outcome space. The loss function is formulated as

$$L_{1}=\sum_{i=1}^{n}p\left(f_{\phi_{i},a_{i}}^{1}(U)=y_{i}\right).$$

Stage 2 (Adjusting for hidden confounding): Since the learned representation *ϕ* may not satisfy unconfoundedness due to unmeasured confounding, we introduced a second CNF $f_{\phi ,\text{a}}^{2}$. It transforms a new latent variable $\tilde{U}\sim N(0,I)$ to an interventional latent variable that approximates *p*(*u*|*ϕ*). The resulting latent sample is then passed through the Stage 1 transformation $f_{\phi ,\text{a}}^{1}$, which shifts the latent distribution *p*(*u*|*ϕ*, *a*) toward the interventional distribution *p*(*u*|*ϕ*). This adjustment allows us to account for hidden biases arising from factors like clinician decision-making, treatment selection bias, and unobserved patient severity. The loss function is formulated as

$$L_{2}=\overset{n}{\underset{i=1}{\sum }}p\left(f_{\phi_{i},a_{i}}^{1}\left(f_{\phi_{i},a_{i}}^{1}(\tilde{U})\right)=y_{i}\right).$$

Inference stage: During inference, we sampled $\tilde{U}$ using the CNF from Stage 2 and mapped it to *y* using the CNF from stage 1, then averaged multiple predicted outcomes. ITE was obtained by the difference between the averaged outcomes under NIV and under HFNC.

We split the UCSD early HFNC/NIV cohort into an 80% training and 20% internal validation set. We applied Bayesian optimization[22] to tune hyperparameters including learning rates, weight regularization factor, number of hidden layers and flow depth. The optimization process was guided by validation performance across the CFR model and both CNF modules. Each module was trained separately using the Adam optimizer[23], with early stopping based on validation loss to mitigate overfitting. Model performance, including both predictive accuracy and ITE estimation quality, was evaluated on the entire UCSD early HFNC/NIV cohort. To assess generalizability, we further conducted external validation on an independent early HFNC/NIV cohort from UCI.

The predicted Individual Treatment Effect (ITE) was defined as the difference between the predicted probability of IMV under NIV and under HFNC, when each was given as the first intervention following the VentNet T0 timepoint. To assess predictive performance, we reported two standard metrics for IMV prediction: the Area Under the Receiver Operating Characteristic Curve (AUC) and the Area Under the Precision-Recall Curve (PR-AUC). ITE estimation quality was further evaluated by examining patient outcomes under treatment concordance. As baseline comparisons, we included commonly used data-driven ITE estimation methods in clinical settings, including Causal Forest[15], X-Learner[16], and the CFR model.

## Supplementary Files

This is a list of supplementary files associated with this preprint. Click to download.

- Supplementarymaterial.docx

## Figures and Tables

**Table 1.** Baseline characteristics of patients in UCSD and UCI early HFNC/NIV cohorts.

Variable	UCSD (development site)		UCI (validation site)	
	NIV-treated	HFNC-treated	NIV-treated	HFNC-treated
**Characteristic**				
Encounters, N	591	1365	38	131
Age(years), mean (SD)	63(16.3)	61(16.6)	66(15.0)	63(17.6)
**Gender, N (%)**				
Male	369(62.4)	806(59.0)	20(52.6)	86(65.6)
**Organ dysfunction, median (IQR)**				
**Charlson Comorbidity Index, Median (IQR)**	3.0(1.0–5.0)	2.0(1.0–5.0)	3.0(1.0–6.0)	2.0(0.0–3.0)
Congestive Heart Failure Component, N (%)	245(41.5)	315(23.1)	15(39.5%)	29(22.1)
Chronic Obstructive Pulmonary Disease, N (%)	161(27.2)	234(17.1)	4(10.5%)	8(6.1)
SOFA^c^ score (at Vent.io T0), Median (IQR)	1.0(0.0–3.0)	1.0(0.0–3.0)	2.0(1.0–4.0)	1.0(0.0–3.0)
**Outcomes, N (%)**				
IMV	122 (20.6)	334 (24.5)	9 (23.7)	38 (29.0)
Mortality	147 (24.9)	431(31.6)	9 (23.7)	29 (22.1)
Hospice	6 (1.0)	11(0.8)	5 (13.2)	31 (23.7)

* The HFNC-treated and NIV-treated refer to the actual initial treatments administered to patients after the Vent.io T0 timepoint.

a SOFA: Sequential Organ Failure Assessment.

b We counted the number of patients for each initial ventilation including both its individual use and any combined usage methods categorized under the same category.

**Table 2.** Baseline characteristics of patients in UCSD and UCI early HFNC/NIV cohorts by the predicted individualized treatment effect.

Variable	UCSD (development site)				UCI (validation site)			
	NIV preferred	HFNC preferred	Indifferent	*P*^c^ value	NIV preferred	HFNC preferred	Indifferent	*P* value
**Characteristic**								
Encounters, N	1296	465	195	-	50	20	99	-
Age(years), mean (SD)	62(16.9)	62(16.3)	64(16.7)	<.001	61(14.5)	65(20.1)	65(17.7)	.947
**Gender, N (%)**								
Male	796(61.4)	260(55.9)	119(61.0)	-	34(68.0)	11(55.0.0)	61(61.6)	-
**Organ dysfunction**								
**Charlson Comorbidity Index, Median (IQR)**	2.0(1.0–5.0)	3.0(1.0–5.0)	3.0(1.0–6.0)	.035	2.5(1.0–5.5)	2.0(1.0–3.0)	2.0(0.5–3.0)	.358
Congestive Heart Failure Component, N (%)	364(28.1)	132(28.4)	64(32.8)	.255	30(30.3)	9(18.0)	5(25.0)	.269
Chronic Obstructive Pulmonary Disease, N (%)	250(19.3)	98(21.1)	47(24.1)	.391	7(7.1)	4(8.0)	1(5.0.)	.907
SOFA score (at Vent.io T0), Median (IQR)	1.0(0.0–3.0)	1.0(0.0–3.0)	1.0(0.0–3.0)	.703	1.0(0.0–3.0)	1.5(1.0–4.0)	1.0(0.0–3.0)	.330
**Baseline physiology** ^a^								
Heart Rate, median (IQR), beats/min	102(90–105)	101(88–114)	102(91–116)	.549	102(85–116)	108(87–123)	98(84–116)	.734
> 80, N (%)	1113(85.9)	394(84.7)	171(87.7)	.602	43(86.0)	16(80.0)	80(80.8)	.708
Temperature, median (IQR), °c	36.9(37.0–37.3)	36.8(36.0–37.2)	36.8(37.0–37.1)	.055	36.7(37.0–37.1)	36.7(37.0–37.1)	36.7(36.0–37.1)	.678
< 36, N (%)	58(4.5)	28(6.0)	11(5.6)	.377	1(2.0)	1(5.0)	8(8.1)	.326
> 38, N (%)	87(4.5)	45(2.3)	72(3.7)	.458	3(6.0)	3(15.0)	6(6.1)	.342
Mean Arterial Pressure, median (IQR), mm Hg	80.0(70.0–91.0)	80.5(71.0–90.0)	78.8(69.0–89.0)	.285	83.0(77.0–93.0)	86.0(74.0–93.2)	83.0(73.0–95.0)	.966
< 65, N (%)	163(12.6)	64(13.8)	31(15.9)	.405	1(2.0)	2(10.0)	6(6.1)	.355
Respiratory Rate, median (IQR), breaths/min	26(22–29)	26(22–29)	26(23–30)	.072	29(26–34)	26(23–30)	28(25–33)	.292
> 20, N (%)	1121(86.5)	390(83.9)	174(89.2)	.157	48(96.0)	18(90.0)	91(91.9)	.569
PaCO2, median (IQR), mm Hg	38(32–47)	37(31–48)	38(30–44)	.321	36(33–40)	34(32–40)	37(32–44)	.715
**Interventions after Vent.io T0**^b^ **N (%)**								
Steroids administration	403(31.1)	120(25.8)	59(30.3)	.054	24(48.0)	10(50.0)	55(55.6)	.945
Antibiotics administration	1056(81.5)	373(80.2)	169(86.7)	.062	43(86.0)	19(95.0)	74(74.7)	.031
Vasopressors administration	399(30.8)	147(31.6)	50(25.6)	.164	15(30.0)	6(30.0)	29(29.3)	.673
Diuretics administration	747(57.6)	251(54.0)	120(61.5)	.117	24(48.0)	10(50.0)	55(55.6)	.231
**Initial respiratory support after Vent.io T0 (N %)**								
HFNC	911(70.3)	319(68.6)	135(69.2)	-	38(76.0)	15(75.0)	78(78.8)	-
**Outcomes (N %)**								
IMV	327(25.2)	91(19.6)	38(19.5)	<.019	22(37.9)	16(32.0)	3(15.0)	.353
Mortality	387(29.9)	129(27.7)	62(31.8)	.532	9(18.0)	4(20.0)	25(25.3)	.582
Hospice	12(0.9)	4(0.9)	1(0.5)	.845	14(28.0)	1(5.0)	21(21.2)	.105
**Average treatment effect, mean (SD)**	−0.024 (0.023)	0.008 (0.008)	0.000 (0.0006)	<.001	−0.054 (0.098)	0.003 (0.001)	0.000 (0.0001)	<.001

a Baseline physiology was measured at the time of Vent.io T0.

b We focused on interventions administered from Vent.io T0 to one hour before ICU discharge for the control group (those not intubated), and from Vent.io T0 to one hour before the time of intubation for the positive group (those who require intubation).

c Testing for the difference *P* value were χ^2^ for categorical variables and Kruskal-Wallis rank-sum test for continuous variables.

**Table 3.** Multivariable Logistic Regression Results (Odds Ratios and p-values) for Predicting the Need for IMV Across Methods and Sites.

Method	Site	NIV concordance	HFNC concordance	Age	Gender	CCI score	SOFA score	Vent.io score
**Causal Forest**	UCSD	**0.443** **p<.001**	**0.778** **p=.033**	0.988 p<.001	1.046 p=.685	0.929 p<.001	1.044 p=.101	1.276 p=.083
	UCI	0.415 p=.153	0.903 p=.786	0.994 p=.559	1.046 p=.903	1.089 p=.165	1.051 p=.494	1.032 p=.949
**X-learner**	UCSD	**0.109** **p<.001**	**0.356** **p<.001**	0.984 p<.001	1.035 p=.769	0.932 p<.001	1.066 p=.021	1.267 p=.105
	UCI	0.409 p=.089	0.731 p=.397	1.012 p=.239	0.723 p=.337	0.971 p=.622	1.094 p=.199	2.329 p=.079
**CFR**	UCSD	**0.185** **p<.001**	1.846 p<.001	0.992 p=.033	1.039 p=.745	0.949 p=.011	1.020 p=.475	1.294 p=.077
	UCI	0.495 p=.245	0.897 p=.783	0.992 p=.458	1.042 p=.912	1.069 p=.276	1.038 p=.606	1.015 p=.977
**RepFlow-CFR**	UCSD	**0.661** **p=.0049**	**0.677** **p=.019**	0.988 p<.001	1.003 p=.980	0.933 p<.001	1.047 p<.089	1.143 p<.350
	UCI	0.482 p=.373	0.243 p=.121	0.978 p=.252	0.554 p=.328	0.988 p=.901	1.092 p=.424	1.404 p=.747

**Table 4.** Multivariable Logistic Regression Results (Odds Ratios and p-values) for Predicting Mortality & Hospice Across Methods and Sites.

Method	Site	NIV concordance	HFNC concordance	Age	Gender	CCI score	SOFA score	Vent.io score
**Causal Forest**	UCSD	**0.663** **p=.009**	1.040 p=.723	1.020 p<.001	0.844 p=.104	0.955 p=.010	1.260 p<.001	1.245 p<.100
	UCI	0.832 p=.707	0.712 p=.333	1.014 p=.155	0.706 p=.296	0.984 p=.775	1.092 p=.202	2.086 p=.120
**X-learner**	UCSD	**0.592** **p<.001**	1.034 p=.775	1.021 p<.001	0.855 p=.136	0.959 p=.019	1.251 p<.001	1.249 p<.097
	UCI	**0.327** **p=.043**	0.774 p=.492	1.014 p=.182	0.591 p=.127	0.984 p=.777	1.118 p=.115	1.941 p=.163
**CFR**	UCSD	**0.666** **p=.003**	1.176 p=.217	1.022 p<.001	0.831 p=.080	0.960 p=.026	1.237 p<.001	1.274 p=.071
	UCI	0.409 p=.089	0.731 p=.397	1.011 p=.239	0.723 p=.337	0.971 p=.622	1.094 p=.199	2.329 p=.079
**RepFlow-CFR**	UCSD	**0.679** **P=.0049**	0.749 P=.063	1.020 P<.001	0.884 P=.265	0.953 P=.011	1.278 P<.001	1.213 P=.160
	UCI	**0.092** **P=.020**	**0.088** **P=.012**	1.021 P=.292	0.726 P=.608	1.103 P=.295	1.368 P=.010	3.027 P=.276

## Data Availability

Data supporting the findings of this study can be provided upon reasonable request to the corresponding author.

## Code availability:

Code used in this study can bemade available upon reasonable request to the corresponding author.

## References

[1] Lam JY, Lu X, Shashikumar SP, Development, deployment, and continuous monitoring of a machine learning model to predict respiratory failure in critically ill patients. JAMIA Open. 2024;7:ooae141. 39664647 10.1093/jamiaopen/ooae141 PMC11633942

[2] Doshi P, Whittle JS, Bublewicz M, High-velocity nasal insufflation in the treatment of respiratory failure: A randomized clinical trial. Ann Emerg Med. 2018;72:73–83.e5. 29310868 10.1016/j.annemergmed.2017.12.006

[3] Frat J-P, Thille AW, Mercat A, High-flow oxygen through nasal cannula in acute hypoxemic respiratory failure. N Engl J Med. 2015;372:2185–96. 25981908 10.1056/NEJMoa1503326

[4] Azevedo JR, Montenegro WS, Leitao AL, High flow nasal cannula oxygen (hfnc) versus non-invasive positive pressure ventilation (nippv) in acute hypoxemic respiratory failure. a pilot randomized controlled trial. Intensive Care Med Exp. 2015;3:A166.

[5] Grieco DL, Menga LS, Raggi V, Physiological comparison of high-flow nasal cannula and helmet noninvasive ventilation in acute hypoxemic respiratory failure. Am J Respir Crit Care Med. 2020;201:303–12. 31687831 10.1164/rccm.201904-0841OC

[6] Nair PR, Haritha D, Behera S, Comparison of high-flow nasal cannula and noninvasive ventilation in acute hypoxemic respiratory failure due to severe COVID-19 pneumonia. Respir Care. 2021;66:1824–30. 34584010 10.4187/respcare.09130

[7] Grieco DL, Menga LS, Cesarano M, Effect of helmet noninvasive ventilation vs high-flow nasal oxygen on days free of respiratory support in patients with COVID-19 and moderate to severe hypoxemic respiratory failure: The HENIVOT randomized clinical trial: The HENIVOT randomized clinical trial. JAMA. 2021;325:1731–43. 33764378 10.1001/jama.2021.4682 PMC7995134

[8] Coudroy R, Frat J-P, Ehrmann S, High-flow nasal oxygen alone or alternating with non-invasive ventilation in critically ill immunocompromised patients with acute respiratory failure: a randomised controlled trial. Lancet Respir Med. 2022;10:641–9. 35325620 10.1016/S2213-2600(22)00096-0

[9] Tan D, Wang B, Cao P, High flow nasal cannula oxygen therapy versus non-invasive ventilation for acute exacerbations of chronic obstructive pulmonary disease with acute-moderate hypercapnic respiratory failure: a randomized controlled non-inferiority trial. Crit Care. 2024;28:250. 39026242 10.1186/s13054-024-05040-9 PMC11264824

[10] Munroe ES, Prevalska I, Hyer M, High-flow nasal cannula versus noninvasive ventilation as initial treatment in acute hypoxia: A propensity score-matched study. Crit Care Explor. 2024;6:e1092. 38725442 10.1097/CCE.0000000000001092 PMC11081605

[11] Hao J, Liu J, Pu L, High-flow nasal cannula oxygen therapy versus non-invasive ventilation in AIDS patients with acute respiratory failure: A randomized controlled trial. J Clin Med. 2023;12. doi: 10.3390/jcm12041679 PMC9967185 36836213

[12] Rochwerg B, Brochard L, Elliott MW, Official ERS/ATS clinical practice guidelines: noninvasive ventilation for acute respiratory failure. Eur Respir J. 2017;50. doi: 10.1183/13993003.02426-2016 28860265

[13] Oczkowski S, Ergan B, Bos L, ERS clinical practice guidelines: high-flow nasal cannula in acute respiratory failure. Eur Respir J. 2022;59:2101574. 34649974 10.1183/13993003.01574-2021

[14] Ding P. The central role of the propensity score in observational studies for causal effects. A First Course in Causal Inference. Boca Raton: Chapman and Hall/CRC 2024:137–50.

[15] Wager S, Athey S. Estimation and inference of heterogeneous treatment effects using random forests. J Am Stat Assoc. 2018;113:1228–42.

[16] Künzel SR, Sekhon JS, Bickel PJ, Metalearners for estimating heterogeneous treatment effects using machine learning. Proc Natl Acad Sci U S A. 2019;116:4156–65. 30770453 10.1073/pnas.1804597116 PMC6410831

[17] Rubin DB. Estimating causal effects of treatments in randomized and nonrandomized studies. J Educ Psychol. 1974;66:688–701.

[18] Shalit U, Johansson FD, Sontag D. Estimating individual treatment effect: generalization bounds and algorithms. arXiv [stat.ML]. 2016.

[19] Melnychuk V, Frauen D, Feuerriegel S. Causal Transformer for Estimating Counterfactual Outcomes. arXiv [cs.LG]. 2022.

[20] Melnychuk V, Frauen D, Feuerriegel S. Bounds on representation-induced confounding bias for treatment effect estimation. arXiv [stat.ML]. 2023.

[21] Durkan C, Bekasov A, Murray I, Neural Spline Flows. arXiv [stat.ML]. 2019.

[22] Snoek J, Larochelle H, Adams RP. Practical Bayesian optimization of machine learning algorithms. arXiv [stat.ML]. 2012.

[23] Kingma DP, Ba J. Adam: A method for stochastic optimization. arXiv [cs.LG]. 2014.

[24] den Hengst F, Otten M, Elbers P, Guideline-informed reinforcement learning for mechanical ventilation in critical care. Artif Intell Med. 2024;147:102742. 38184349 10.1016/j.artmed.2023.102742

